# P-1343. Extended Spectrum Beta Lactamases (ESBL) and New Delhi Metallo Protein (NDM) genes co-occurrence in Phenotypic ESBL Enterobacteriaceae (ESBL-E) isolates causing community-acquired pyelonephritis

**DOI:** 10.1093/ofid/ofae631.1520

**Published:** 2025-01-29

**Authors:** Deepak Kumar, Lochan Khandelwal, Gopal Krishna Bohra, Vibhor Tak, Durga Shankar Meena, Naresh Kumar Midha, M K Garg, Vidhi Jain, Gautam Ram Choudhary

**Affiliations:** All India Institute of Medical Sciences, Jodhpur (India), Jodhpur, Rajasthan, India; AIIMS Jodhpur, Jodhpur, Rajasthan, India; AIIMS, Jodhpur, Rajasthan, India; AIIMS Jodhpur, Jodhpur, Rajasthan, India; AIIMS, Jodhpur, Rajasthan, India; AIIMS, Jodhpur, Rajasthan, India; AIIMS, Jodhpur, Rajasthan, India; AIIMS, Jodhpur, Rajasthan, India; AIIMS Jodhpur, Jodhpur, Rajasthan, India

## Abstract

**Background:**

Multidrug resistance Enterobacteriaceae causing community-acquired pyelonephritis is an emerging threat; most isolates are EBSL, as per Indian data. After the results of the MERINO trial, most guidelines favors Carbapenem for treating community-acquired pyelonephritis. Overuse of carbapenem may lead to Carbapenem resistance Enterobacteriaceae (CRE) in the community. We analyzed the ESBL and CRE genes in the clinically significant urinary isolates with phenotypic ESBL -E from patients with community-acquired pyelonephritis.

**Table 1:**
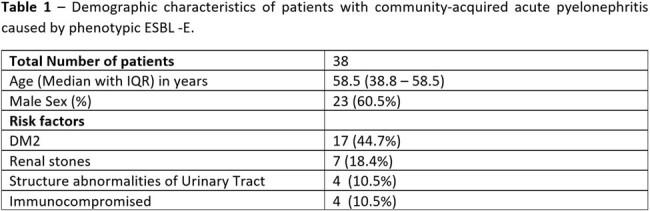
Demographic characteristics of patients with community-acquired acute pyelonephritis caused by phenotypic ESBL -E.

**Methods:**

Patients with a diagnosis of community-acquired acute pyelonephritis with age ≥ 18 years and urine culture showing the growth of Enterobacteriaceae with phenotypic ESBL-E are included in the study. If urine culture grew more than one organism in Enterobacteriaceae, other GNBs were excluded. The phenotypic ESBL detection was based on resistance to a third-generation cephalosporin (Cefotaxime, Cefpodoxime, Ceftazidime) and a monobactam (Aztreonam). These positive isolates were further processed using the combination disk method. A ≥5mm increase in zone diameter for either antimicrobial agent tested in combination with clavulanate vs the zone diameter of the agent when tested alone was considered ESBL (Clinical and Laboratory Standards Institute (CLSI) *Performance standards for antimicrobial susceptibility tests)*. Simultaneously, multiplexed Real-time PCR (TRUPCR® UTI AST Panel Kit, Europe) was done to detect ESBL genes (CTX-M, TEM, SHV) and CRE genes (OXA-48, KPC, NDM, VIM, IMP) in these isolates.

**Table 2:**
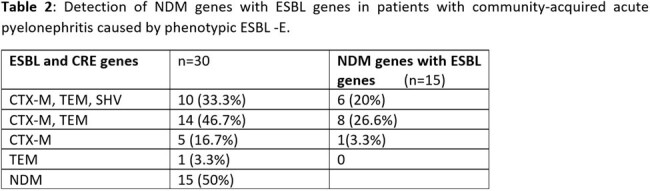
Detection of NDM genes with ESBL genes in patients with community-acquired acute pyelonephritis caused by phenotypic ESBL -E.

**Results:**

A total of 38 isolates with phenotypic ESBL in patients diagnosed with community-acquired pyelonephritis were processed for RT-PCR, and 30 were detected with ESBL and CRE genes. The demographic and clinical characteristics are shown in Table 1. The most common ESBL gene was CTX-M 29 (.96.7%) followed by TEM 25 (83.3%). Multiple ESBL genes were detected in 24(80%) isolates. Among CRE genes, the NDM genes were detected in 15 (50%) isolates with other ESBL genes (Table 2).

**Conclusion:**

The presence of CRE genes in phenotypic ESBL -E in community-acquired acute pyelonephritis could be due to the injudicious use of carbapenem. This may imply that CRE will be spread in the community in the future, making it difficult to manage this infection.

**Disclosures:**

**All Authors**: No reported disclosures

